# The Role of Capsid in HIV-1 Nuclear Entry

**DOI:** 10.3390/v13081425

**Published:** 2021-07-22

**Authors:** Anabel Guedán, Eve R. Caroe, Genevieve C. R. Barr, Kate N. Bishop

**Affiliations:** Retroviral Replication Laboratory, The Francis Crick Institute, London NW1 1AT, UK; anabel.guedan@crick.ac.uk (A.G.); eve.caroe@crick.ac.uk (E.R.C.); genevieve.barr@crick.ac.uk (G.C.R.B.)

**Keywords:** HIV-1, capsid, core, nuclear entry, NPC, uncoating

## Abstract

HIV-1 can infect non-dividing cells. The nuclear envelope therefore represents a barrier that HIV-1 must traverse in order to gain access to the host cell chromatin for integration. Hence, nuclear entry is a critical step in the early stages of HIV-1 replication. Following membrane fusion, the viral capsid (CA) lattice, which forms the outer face of the retroviral core, makes numerous interactions with cellular proteins that orchestrate the progress of HIV-1 through the replication cycle. The ability of CA to interact with nuclear pore proteins and other host factors around the nuclear pore determines whether nuclear entry occurs. Uncoating, the process by which the CA lattice opens and/or disassembles, is another critical step that must occur prior to integration. Both early and delayed uncoating have detrimental effects on viral infectivity. How uncoating relates to nuclear entry is currently hotly debated. Recent technological advances have led to intense discussions about the timing, location, and requirements for uncoating and have prompted the field to consider alternative uncoating scenarios that presently focus on uncoating at the nuclear pore and within the nuclear compartment. This review describes recent advances in the study of HIV-1 nuclear entry, outlines the interactions of the retroviral CA protein, and discusses the challenges of investigating HIV-1 uncoating.

## 1. Introduction

**Figure 1:**
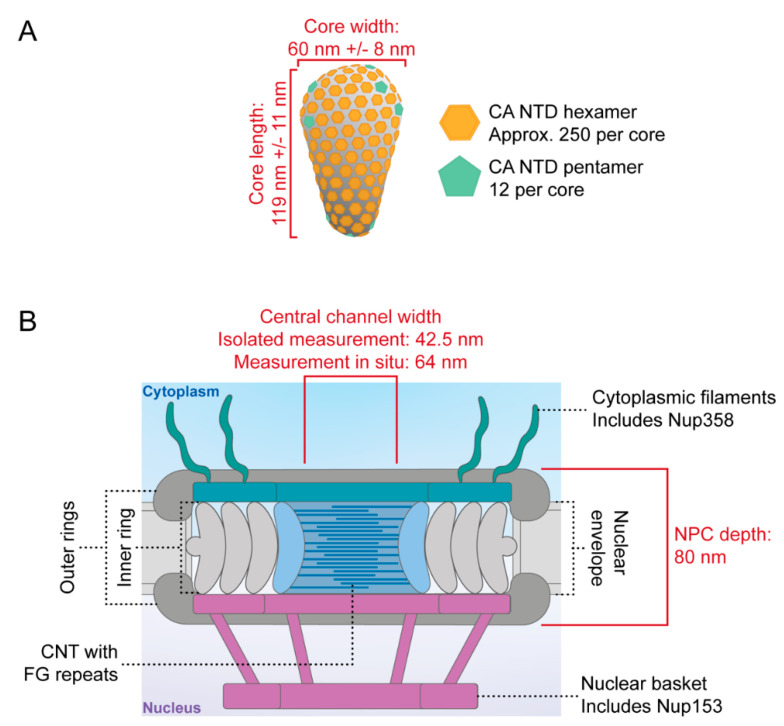
The HIV-1 core and the human NPC. (**A**) Diagram illustrating how CA assembles to form the fullerene shape of the HIV-1 core, where CA N-terminal domain hexamers are shown in orange and CA N-terminal domain pentamers are shown in green. Core measurements shown in red are taken from [7]. (**B**) Diagram of the human NPC highlighting relevant features, labelled with dashed lines. Pore measurements shown in red are taken from [35,36]. CNT = channel nucleoporin heterotrimer; FG repeats = phenylalanine-glycine repeats.

The retroviral capsid (CA) protein is critical for successful progression of the virion through the early post-entry stages of replication, which include the processes that occur between host cell entry and integration. The CA protein consists of an N-terminal domain (NTD) and a C-terminal domain (CTD) separated by a flexible linker [1,2,3,4]. NTDs of adjacent CA monomers make interactions to form hexameric or pentameric rings of CA. The CTDs make dimeric interactions to join the CA rings into a lattice of ~250 hexamers with 12 pentamers [5,6]. The inclusion of pentamers induces curvature to form a closed CA core. Their placement in the lattice differs between retroviral genera, giving rise to distinctive morphologies. The HIV-1 core has a fullerene cone shape [7] due to the inclusion of 5 pentamers at the narrow end and 7 at the wide end of the cone [1,8,9,10] (Figure 1A).

Following fusion of the viral envelope with the host cell membrane, the viral core is released into the cytoplasm. The core consists of the viral genetic material, bound by the nucleocapsid (NC) protein, and the viral enzymes, reverse transcriptase (RT) and integrase (IN), all enclosed by the protective CA lattice. RT and IN mediate the essential processes that define retroviral replication: reverse transcription and integration, respectively. Reverse transcription is the process by which the incoming viral positive sense, single-stranded RNA genome is converted into double-stranded DNA, while integration involves the insertion of this retroviral cDNA into host cell chromatin, to form a provirus. At some point before integration, the CA shell is thought to be degraded or lose integrity, which is referred to as uncoating. However, we still do not have a precise definition of uncoating, and whether complete breakdown of the capsid lattice occurs or whether the lattice “opens” to release the viral nucleic acid is unclear.

Early work suggested that the capsid shell of HIV was lost early after cell entry (reviewed in [11]). Biochemical analysis of pre-integration complexes (PICs) found little CA present [12] and particles were only sensitive to tripartite motif-containing protein 5α (TRIM5α), a host restriction factor that targets the CA lattice, for approximately an hour after cell entry [13]. Furthermore, uncoating was shown to be dependent on reverse transcription [14,15,16,17,18], which was believed to mainly take place in the cytoplasm, as integration-competent PICs could be isolated from cytoplasmic fractions [19,20,21,22]. Using GFP as a fluid phase marker in virions, and following infections by live-cell imaging, Mamede et al. endorsed an early uncoating model. They reported a partial loss of GFP signal upon membrane fusion, presumably representing loss of GFP from between the core and the viral membrane, followed by a second signal loss shortly afterwards in the cytoplasm, interpreted as loss of core integrity and, thus, uncoating [18]. However, the same lab has also shown that HIV-1 trafficked to the cell nucleus via microtubules and that this was dependent on CA [23]. Therefore, it seemed likely that some CA was present up until the viral complex reached the nucleus. In addition, the CA lattice has been reported to protect HIV-1 cDNA from DNA sensing in the cytoplasm [24,25,26], which could otherwise lead to immune system activation [27]. Indeed, HIV-1 appears to require a CA lattice with optimal stability and flexibility [6,10,28]. Studies with unstable or hyper-stable CA mutants have shown that early uncoating or the inability to uncoat to WT levels has detrimental effects on viral infectivity [26,28,29].

More recent studies have suggested that the core reaches the nucleus earlier than previously thought [30,31,32], and the prevailing opinion is that the core is mostly intact at this point. Many retroviruses, such as murine leukaemia virus (MLV), must wait for mitosis and the breakdown of the nuclear envelope (NE) before they can access the host chromatin. However, HIV-1 can infect non-dividing cells and thus must be able to pass through a nuclear pore. Some years ago, the CA protein was identified as the critical determinant for nuclear entry via the nuclear pore, supporting the notion that CA is present in the complex at nuclear entry [33,34]. As HIV-1 infection is independent of the cell cycle and infectivity is similar in aphidicolin-treated and untreated cells [34], it seems likely that HIV-1 nuclear entry occurs via nuclear pores even in cycling cells. However, whether/how the CA is assembled at the point of nuclear entry is currently hotly debated. This review will discuss the various proposals for what happens to the core once it reaches the nucleus and how cellular factors influence translocation across the nuclear pore.

## 2. The Nuclear Pore as a Barrier

Nuclear pore complexes (NPCs) consist of repeated assemblies of ~30 different nucleoporins and constitute one of the largest macromolecular complexes in cells (Figure 1B). NPCs stud the lipid membrane that surrounds the nucleus and mediate nucleocytoplasmic transport, where small molecules can diffuse passively and larger macromolecules can be actively carried by nuclear transport factors (reviewed in [35]). Originally, the HIV-1 core was thought to be too large to pass through the NPC. Structural studies determined that the viral core is ~60 nm in width [7,10] (Figure 1A), which exceeded the ~40 nm inner diameter of pores measured from NPCs in isolated nuclear envelopes [37] (Figure 1B). Thus, it seemed reasonable that the core must disassemble before the viral cDNA entered the nucleus. Whether the uncoating process started in the cytoplasm en route to the nucleus or began at the pore itself was debated, with data supporting both models presented in the literature [18,38,39,40,41,42,43,44]. For example, Francis et al. developed a live-cell imaging system, where a DsRed-labelled CypA was packaged into virions through the high-avidity binding to CA and uncoating, as measured by loss of CypA-DsRed, was observed at the NPC, with a small amount of CA remaining in association with the PIC in the nucleus [41,42,44]. However, in these experiments, loss of DsRed might also have reflected displacement of the tagged CypA with other CA-binding factors at the pore rather than true uncoating. Although many groups believed that the core must disassemble to pass through the NPC, it was agreed that some CA must be present in the nucleus because CA interacted with a nuclear factor called cleavage and polyadenylation factor 6 (CPSF6) [45,46,47,48] and influenced integration site selection [32,49,50,51,52]. Where this CA came from and how it remained with the viral cDNA were not clear. Interestingly, recently, the cellular environment has been found to influence the conformation of NPCs under certain circumstances [53,54], indicating that the pores may be more flexible than originally thought. With this in mind, Zila et al. used correlative electron microscopy (CLEM) and cryo-electron tomography (cryo-ET) to analyse the diameter of the central channel of NPCs in SupT1-R5 cells infected with HIV-1 and found that it was ~64 nm in width, which would make it geometrically possible for an intact core to pass through the pore [36] (Figure 1B). Excitingly, they were also able to visualise intact cores near and inside the NPC, with a tendency for the pentamers at the narrow end of the core to point towards the centre of the NPC channel. However, the structures they detected inside the nucleus did not resemble the intact cores at the NPC, suggesting that some sort of remodelling event modified the core at the NPC or after delivery into the nucleus [36]. The limitation of this study is that most of the data were obtained with a CPSF6-binding deficient CA that may not uncoat in an identical manner to wild type virus. In a more recent publication from the same lab, the authors used WT HIV-1 and detected conical and elongated structures that appeared to be open viral cores inside the nucleus of HeLa derived cells [55].

## 3. Core Remodelling at the NPC

The concept of core remodelling at nuclear entry was first introduced by Blanco-Rodriguez et al. [56]. By combining immunoelectron microscopy to follow CA and the ANCHOR system to track the viral cDNA, they observed by CLEM a complex of viral cDNA decorated by multiple CA proteins. During and after nuclear entry in lymphocytes, their staining pattern changed to resemble a “pearl necklace-like” configuration [56]. The observation of divergent CA arrangements before and after nuclear entry suggested that interactions with the NPC caused structural changes to the CA lattice. In addition, we have recently shown that HIV-1 mutants with hyper-stable CA lattices are unable to integrate because they are stalled at nuclear entry, potentially trapped at the NPC. This indicates that a lack of CA lattice flexibility at nuclear entry is detrimental for viral infectivity [57]. Together, these observations suggest that a remodelling event is required at the NPC to grant nuclear entry. Another recent study using GFP-CA HIV-1 as well as a GFP fluid phase marker to monitor core disassembly and integrity, respectively, suggested that HIV-1 uncoats very rapidly after loss of core integrity, shortly before integration near the site of integration itself. Although they implied that an intact core enters the nucleus, the authors pointed out that a degree of CA lattice flexibility or core remodelling might be needed to pass through the NPC [58].

## 4. CA in the Nucleus

**Figure 2:**
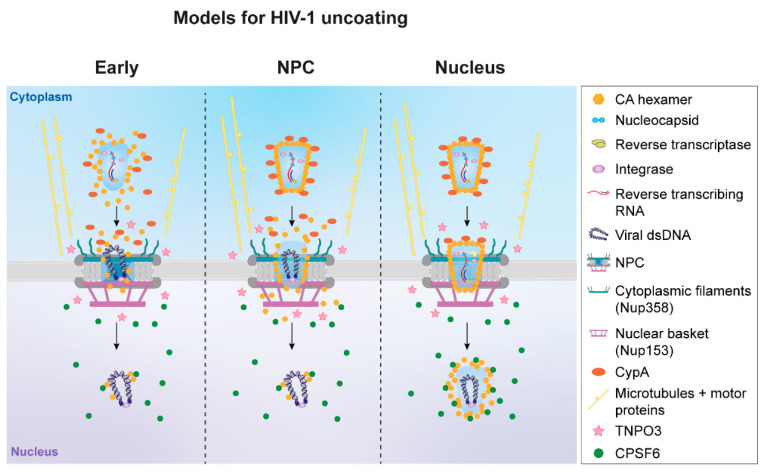
Models for HIV-1 uncoating. HIV-1 uncoating has been proposed to occur either early in the cytoplasm, at the NPC or after nuclear entry. The three possibilities are depicted, with host factors involved in uncoating and nuclear entry illustrated. Figure adapted from [57].

Various groups have now reported the presence of CA in the nucleus by fixed immunofluorescence microscopy [32,43,48,49,55,56,59,60,61]. Muller et al. have recently shown efficient detection of nuclear CA signals in HeLa-derived cells is improved by fixing with methanol or by displacing CPSF6 from subviral complexes using PF74 treatment after nuclear entry has occurred [55]. In addition, treatment with protease after fixation and permeabilisation, was shown to enhance nuclear CA signal in HeLa-T4 cells [49]. However, it is not possible to know what the oligomeric state of CA is in these experiments, nor whether these complexes will lead to productive infection. Other groups have shown the presence of CA multimers in the nucleus biochemically by subcellular fractionation [32,57], but again, these experiments cannot determine whether the whole CA lattice is present. Recently, live-cell imaging of labelled CA protein has been performed. Burdick et al. reported successful incorporation of GFP-CA by making mixed particles with a 1:15 ratio of GFP-CA to unlabeled-CA. In these experiments, no detectable loss of GFP was observed until the core reached the integration site [30]. However, as only a fraction of the CA was labelled with GFP, it is possible that this does not represent a full CA lattice. Li et al. very recently combined GFP-CA labelling with the GFP fluid phase marker and, in contrast to a previous report [18], found that cores retained integrity until just before integration, suggesting that intact cores were transported through the NPC [58]. This is supported by recent CLEM experiments that visualised CA assemblies around NPCs, and what looked like intact cores within the nucleus [55]. These cores were shown to contain viral cDNA as labelled using the ANCHOR system [56], leading the authors to propose that uncoating happens inside the nucleus by localised physical disruption instead of by cooperative disassembly [55]. It is worth noting, however, that in this case, the ANCH sequence-containing viral cDNA must be accessible to the OR-GFP protein, suggesting a partial opening of the CA lattice. The debate around the oligomeric state of CA in the nucleus continues. We have summarised the three main hypotheses regarding the timing of uncoating in Figure 2.

## 5. Nuclear Entry and Reverse Transcription

Traditionally, reverse transcription was thought to complete in the cytoplasm prior to nuclear entry as integration-competent PICs could be isolated from cytoplasmic fractions [19,20,21,22]. Additionally, premature uncoating triggered anti-viral responses due to exposure of viral cDNA to cytoplasmic sensors [24,25,26,27]. Importantly, uncoating was shown to be dependent on reverse transcription [14,15,16,17,18]. As uncoating was thought to occur before nuclear entry, this meant that reverse transcription must also occur before this step. In vitro studies using atomic force microscopy (AFM) suggested that reverse transcription increases the pressure inside the core triggering uncoating [17]; this agrees with our previous assessment that uncoating is initiated after first strand transfer during reverse transcription [16]. Rankovic et al. have recently suggested that different stages of reverse transcription induce mechanical changes in the capsid that progressively remodel the viral core to prime it for uncoating [62]. By using an impressive in vitro replication system, Christensen et al. imaged viral cDNA loops extruding from partially uncoated cores [63]. This also suggested that reverse transcription may occur inside intact, or nearly intact, cores and that synthesis of double-stranded viral cDNA induced physical changes to the lattice. More recent cell-based studies have confirmed that reverse transcription can occur inside an intact or stabilised core [30,55,57,58,63]. Time-of-addition studies in infected cells showed that sensitivity to RT inhibitors was lost before sensitivity to the CA lattice-binding small molecule, PF74, suggesting that assembled capsids composed of CA hexamers are retained until after completion of reverse transcription [30,31,32]. Recently, with the detection of CA assemblies in the nucleus, there has been growing evidence that, although reverse transcription starts in the cytoplasm (or even in virions), it actually completes in the nucleus in primary cells [30,31,32,40,43,44,55,58,64,65]. By using YFP-tagged APOBEC3F, that binds to PICs, Burdick et al. showed that reverse transcription was not required for PIC nuclear entry [40]; and in a recent elegant study, Dharan et al. blocked passage through nuclear pores and measured viral replication kinetics, concluding that reverse transcription completed after nuclear entry [31]. As some of the studies linking uncoating to reverse transcription, including ours [16], used methods such as sensitivity to TRIM-Cyp as a measure of uncoating, it is possible that they were, in fact, measuring nuclear entry and movement out of the TRIM-Cyp-containing cytoplasmic compartment instead of uncoating. Together, these observations have led to the hypothesis that viral complexes with an intact or almost intact CA lattice, that is still undergoing reverse transcription, may be able to traverse a nuclear pore and enter the nucleus. The fact that reverse transcription could be now considered part of the nuclear events in the HIV-1 replication cycle opens a debate about how this process influences HIV-1 nuclear entry.

## 6. Host Factors That Interact with HIV-1 CA around the Nuclear Pore Complex

As well as multimerising with itself, the HIV-1 CA protein appears to interact with a wider variety of host factors than any other viral protein. The CA lattice presents a large surface area with binding pockets and loops on individual CA monomers, and additional binding sites created by higher order CA assemblies. In the cytoplasm, CA interacts with various microtubule-associated proteins and motor proteins which facilitate nuclear trafficking [23,66,67,68,69] as well as cyclophilin A (CypA) [70,71,72,73]. Importantly, the CA lattice has also been referred to as a pathogen-associated molecular pattern (PAMP) because it is the target for several host restriction factors including TRIM5α [74] and myxovirus resistance protein 2 (MxB) [75,76,77] in the cytoplasm and SUN proteins at the nuclear pore [78]. HIV-1 CA goes on to make multiple interactions with host cell factors at the NPC and has recently been shown to re-localise the nuclear protein CPSF6 into puncta within nuclear speckles [32,43,50,57]. Thus, it is feasible that some or all of these interactions alter the stability and architecture of the CA lattice, facilitating nuclear entry.

### 6.1. Cytoskeleton-Associated Factors

Incoming HIV-1 particles hijack cell cytoskeleton trafficking pathways to traverse the cytoplasm, appearing to alternate between fast, direct pathways and slow ‘wandering’ pathways towards the nucleus, characteristic of microtubule-associated retrograde transport and actin-associated trafficking, respectively [23,79]. Specifically, the HIV-1 core has been shown to modulate trafficking through the cytoplasm by engaging various elements of the opposing microtubule motor protein complexes, dynein and kinesin-1 [23,67,68,80,81,82]. These interactions may have functions beyond trafficking. For example, HIV-1 CA binding to the dynein adaptor protein bicaudal B homolog 2 (BICD2) [82,83] is required to avoid detection by immune sensing mechanisms in macrophages [83], whereas kinesin family member 5A (KIF5A) and KIF5B (components of the kinesin-1 motor complex) were reported to be required for HIV-1 nuclear entry [68] and have been implicated in uncoating [67]. CA-mediated interactions with the host cell cytoskeleton may also contribute to docking of cores at the NPC, with the actin cytoskeleton implicated in perinuclear movement of subviral complexes [79], KIF5B required for nucleoporin 358 (Nup358) relocalisation to the cytoplasm [81], and the recent observation of HIV-1 cores observed associated to microtubules around the NPC [36].

### 6.2. Cyclophilin A

CypA is a peptidyl-prolyl cis-trans isomerase that has been proposed to alter CA lattice stability [84,85,86] and affect reverse transcription and nuclear import [72,73,87]. CypA canonically binds the CypA binding loop in the CA NTD (residues 85–93) [70]. An additional non-canonical CypA binding site has been reported between adjacent hexamers along the axis of the highest curvature, and this has been suggested to stabilise the HIV-1 core [87,88]. CypA is incorporated into immature virions [71,89], however it is the CypA present in the cytoplasm of target cells that is required for HIV-1 infection in certain cell lines, such as Jurkat T cells and primary CD4^+^ T cells [71,73,90]. The CypA literature is somewhat confusing as the effects of inhibiting the CA–CypA interaction either by CypA knock down, addition of the inhibitor cyclosporin A (CsA) or by introducing mutations into CA, have differing effects depending on cell type and virus strain [71,91,92]. Moreover, recently, it has been suggested that CypA binding protects HIV-1 from restriction by human TRIM5α [93,94]. However, disruption of the CA–CypA interaction has been correlated with defects in nuclear entry [72,73], and with reduced dependency on the nuclear pore proteins Nup358 and Nup153 [95,96,97]. Conversely, in Vero cells, CypA was reported to inhibit HIV-1 infection by blocking nuclear entry [98]. Thus, the precise role of CypA in HIV-1 replication is still to be determined.

### 6.3. Transportins

Given the cellular role of transportins in importing proteins into the nucleus, it is perhaps unsurprising that proteins from this family have been implicated in HIV-1 nuclear entry. Transportin 3 (TNPO3 or TRN-SR2) was first identified as essential for HIV-1 integration in siRNA screens [99,100] and has been shown to interact with CA [101,102,103] as well as HIV-1 IN [104]. Moreover, TNPO3 interacts with multiple host cell factors which are involved in HIV-1 nuclear entry such as Nup358 [72], Nup153 [96], and CPSF6 [86,103,105] and may influence their activity. For a review on the role of TNPO3 in HIV-1 nuclear import, see Tabasi et al. [106]. Transportin-1 (TNPO1) has also been reported to bind HIV-1 CA at residue G89 within the CypA binding loop, and it appears to be able to displace CypA binding at the NPC [107].

### 6.4. Nucleoporins

Several of the Nups which compose the NPC were identified as co-factors for HIV-1 infection in siRNA screens [99,100,108,109]. Later, it was shown that HIV-1 CA could bind the phenylalanine-glycine (FG) repeats found in multiple Nups [97] but two Nups have been particularly well characterised with regard to HIV-1 interactions: Nup358 [72,81,110] and Nup153 [46,47,96,111]. Direct interaction with Nups provides a mechanism for how HIV-1 CA directs nuclear entry.

#### 6.4.1. Nup358

Nup358, also known as RanBP2, forms long filaments that project from the NPC into the cytoplasm and is involved in the Ran-GTPase cycle used to shuttle factors between the cytoplasm and nucleus [35,112,113]. Nup358 binds to the CypA binding loop in CA, via a cyclophilin homology domain at the C-terminus [72,110,114], which facilitates docking of incoming cores to the NPC [72,110]. Like CypA, Nup358 is an active isomerase, and catalyses cis-trans isomerisation of prolines in HIV-1 CA [114], although the function of this activity is unclear. Interestingly, Dharan et al. showed that HIV-1 infection induces Nup358 to relocate, accumulating in the cytoplasm [81]. This relocalisation appeared to be dependent on CPSF6 and, as mentioned above, is induced by the microtubule-associated factor KIF5B [81].

#### 6.4.2. Nup153

Nup153 forms part of the nuclear basket on the inner leaflet of the NE and it is involved in the quality control and retention of unspliced mRNAs in the nucleus [115,116]. During HIV-1 infection, Nup153 is directly involved in HIV-1 core translocation through the pore and is fundamental to the ability of HIV-1 to infect non-diving cells [95,110,117]. CA was identified as the determinant for the role of Nup153 in HIV-1 nuclear entry via MLV/HIV-1 chimeras, with viruses bearing CA or IN from MLV being insensitive to Nup153 depletion [96]. It was later shown that the CTD of Nup153 contains an FG repeat enriched region, which binds the hydrophobic pocket in the CA NTD [46,47,117]. Additionally, the function of Nup153 is dependent on CypA-CA binding [96].

### 6.5. CPFS6

CPSF6 was initially discovered to interact with HIV-1 CA when a truncated version missing the nuclear localisation signal (CPSF6-358) was identified in a screen for HIV restriction factors [95]. Cytoplasmic CPFS6 binds incoming cores, altering nuclear trafficking on microtubules and reducing infectivity [95,118]. However, full-length endogenous CPSF6 is predominantly nuclear and is recruited to subviral complexes in the nucleus, where it is critical for cores to pass from the nuclear pore to the nuclear interior [48]. It has been proposed that CypA binding to incoming cores prevents premature CPSF6-CA binding in the cytoplasm [118]. HIV-1 infection redirects CPSF6 to puncta in nuclear speckles [32,43,50,57] and CPSF6 is responsible for the integration of HIV into active euchromatin in speckle-associated domains (SPADs) [43,50,52]. Preventing CA from interacting with CPSF6 results in integration into heterochromatin near the NE [51,111] suggesting that CPSF6 is responsible for moving PICs away from the nuclear periphery.

Nup153 and CPSF6 bind to a conserved hydrophobic binding pocket in the CA NTD, involving α-helices 3 and 4 [45,46,47,117]. This pocket also binds the antiretroviral small molecules PF74 [46,119], BI-2 [46], and GS-6207 [120,121]. The CA mutations N74D and A77V disrupt this binding pocket and are widely used to study the function of these host factors. Nup153, CPSF6, and PF74 all make additional contacts with the CTD of an adjacent monomer in a hexamer, across the NTD–CTD interface [46], explaining observations that these ligands bind CA hexamers with >10-fold higher affinity than disassembled CA [47]. This shared Nup153/CPSF6 binding site has led to speculation that these proteins work cooperatively. In fact, Bejarano et al. showed that they bind CA in succession to handover the HIV-1 core from the NPC to the nuclear interior [48]. This also implies that CA is present in a hexameric form when engaged by Nup153 and CPSF6 on the inner leaflet of the NE [46,47].

## 7. Nuclear Entry in Other Retroviruses

Different retroviruses have distinct nuclear entry requirements and this likely reflects the interactions of their CA proteins with cellular factors. König et al. noted that knockdown of Nup358 or Nup153 specifically blocked infection of HIV-1, whereas MLV infection was unaffected, although all genes that influenced integration affected both MLV and HIV-1 [100]. Concordantly, TNPO3 has been shown to be dispensable for MLV infection [99,104]. This is expected as MLV enters the nuclear compartment during mitosis rather than through the NPC. For non-primate lentiviruses, which do traverse nuclear pores, variations in CA-host factor binding may dictate their route of nuclear entry. For example, feline immunodeficiency virus (FIV) CA can bind Nup358 but does not undergo isomerisation, and Nup358 is not required for FIV nuclear entry [114]. Instead, FIV appears to be more reliant on Nup155; interestingly, HIV-1 cores bearing the N74D CA mutant, which prevents binding to Nup153/CPSF6, are similarly reliant on Nup155 for nuclear entry [95]. Equine infectious anaemia virus (EIAV) CA binds Nup153, via an analogous pocket in the CA NTD; however, there are differences in the amino acid side chains within this pocket, which appear to render EIAV resistant to PF74 [117]. Kane et al. showed that both FIV and EIAV are less sensitive to depletion of a range of Nups, including Nup358 and Nup153, than primate lentiviruses [97]. Taken together, with increasing evidence of variation in the host factors HIV-1 can utilise for nuclear entry [72,95,97], this may indicate a range of nuclear entry paths available which are variably exploited by different lentiviruses.

## 8. Challenges of Studying Core Localisation

Currently, there are several models for when and where uncoating occurs (Figure 2). The favoured hypothesis has moved from cytoplasmic uncoating, to uncoating at the NPC, to the most recent reports of uncoating inside the nucleus [18,30,31,42,48,56,61]. These alternative proposals have major implications for the type of viral complex that passes through the NPC. So why is there so much controversy surrounding this step and why have whole cores only recently been reported inside the nucleus? In short, it is difficult to distinguish whole cores from partial shells, and infectious particles from dead-end byproducts. Due to the structural importance of CA in the virion, it is difficult to directly tag the protein, and different staining techniques have resulted in contradictory data. Furthermore, these studies are blighted by the particle to infectivity ratio leading to uncertainty as to whether the viral complexes observed are precursors of successful infection. Additionally, biochemical assays such as cell fractionation also measure the bulk population of CA, not all of which forms part of an infectious complex. Time-of-addition assays with drugs targeting CA or viral enzymes, as well as susceptibility to restriction factors, do measure productive infection, but any conclusions drawn from them rely on assumptions around the method of action of the drugs or restriction factor used. Furthermore, commonly used cell lines may not behave in the same way as natural targets of HIV-1 such as primary CD4^+^ T cells and macrophages, although they are easier to work with. As technologies have improved, more assays are available to study the problem, and there is a greater understanding of what the indirect methods are truly reporting. Hopefully, this will come together in a unifying model for nuclear entry and uncoating in the near future.

## 9. Conclusions and Perspectives

As well as mechanical events that could result in structural changes to the core during passage through the NPC, the binding of different cellular factors, both during and after nuclear entry, likely influences core stability and flexibility. This in turn may affect the lattice structure and its ability to pass through the nuclear pore. Thus, the viral core should be considered as a dynamic structure that binds numerous cellular proteins on its path through the NPC to reach the cellular DNA. The development of new techniques, especially within microscopy, is helping us to learn more about the CA protein and its functions during replication. The critical role of CA in the early stages of HIV-1 replication is reflected in its emergence as a novel antiretroviral target. At least three CA-binding HIV-1 inhibitors have been developed, including PF74, GS-CA1 and GS-6207, which all target the Nup153/CPSF6 binding pocket in the mature CA lattice (reviewed in [122]). In particular, GS-6207 (Lenacapavir) has proved to be a long acting and potent inhibitor of HIV-1 replication and is currently progressing through phase II/III clinical trials (NCT04143594/NCT04150068). A better understanding of the CA protein and its interactions will hopefully allow us to fully define the role of this multifaceted protein in nuclear entry and at other stages of HIV-1 infection.

## Data Availability

Not applicable.

## References

[1] Ganser B.K., Li S., Klishko V.Y., Finch J.T., Sundquist W.I. (1999). Assembly and analysis of conical models for the HIV-1 core. Science.

[2] Du S., Betts L., Yang R., Shi H., Concel J., Ahn J., Aiken C., Zhang P., Yeh J.I. (2011). Structure of the HIV-1 full-length capsid protein in a conformationally trapped unassembled state induced by small-molecule binding. J. Mol. Biol..

[3] Zhao G., Perilla J.R., Yufenyuy E.L., Meng X., Chen B., Ning J., Ahn J., Gronenborn A.M., Schulten K., Aiken C. (2013). Mature HIV-1 capsid structure by cryo-electron microscopy and all-atom molecular dynamics. Nature.

[4] Deshmukh L., Schwieters C.D., Grishaev A., Ghirlando R., Baber J.L., Clore G.M. (2013). Structure and dynamics of full-length HIV-1 capsid protein in solution. J. Am. Chem. Soc..

[5] Pornillos O., Ganser-Pornillos B.K., Kelly B.N., Hua Y., Whitby F.G., Stout C.D., Sundquist W.I., Hill C.P., Yeager M. (2009). X-ray structures of the hexameric building block of the HIV capsid. Cell.

[6] Gres A.T., Kirby K.A., KewalRamani V.N., Tanner J.J., Pornillos O., Sarafianos S.G. (2015). X-ray crystal structures of native HIV-1 capsid protein reveal conformational variability. Science.

[7] Briggs J.A., Wilk T., Welker R., Krausslich H.G., Fuller S.D. (2003). Structural organization of authentic, mature HIV-1 virions and cores. EMBO J..

[8] Ganser-Pornillos B.K., von Schwedler U.K., Stray K.M., Aiken C., Sundquist W.I. (2004). Assembly properties of the human immunodeficiency virus type 1 CA protein. J. Virol..

[9] Pornillos O., Ganser-Pornillos B.K., Yeager M. (2011). Atomic-level modelling of the HIV capsid. Nature.

[10] Mattei S., Glass B., Hagen W.J., Krausslich H.G., Briggs J.A. (2016). The structure and flexibility of conical HIV-1 capsids determined within intact virions. Science.

[11] Campbell E.M., Hope T.J. (2015). HIV-1 capsid: The multifaceted key player in HIV-1 infection. Nat. Rev. Microbiol..

[12] Fassati A., Goff S.P. (2001). Characterization of intracellular reverse transcription complexes of human immunodeficiency virus type 1. J. Virol..

[13] Perez-Caballero D., Hatziioannou T., Zhang F., Cowan S., Bieniasz P.D. (2005). Restriction of human immunodeficiency virus type 1 by TRIM-CypA occurs with rapid kinetics and independently of cytoplasmic bodies, ubiquitin, and proteasome activity. J. Virol..

[14] Hulme A.E., Perez O., Hope T.J. (2011). Complementary assays reveal a relationship between HIV-1 uncoating and reverse transcription. Proc. Natl. Acad. Sci. USA.

[15] Yang Y., Fricke T., Diaz-Griffero F. (2013). Inhibition of reverse transcriptase activity increases stability of the HIV-1 core. J. Virol..

[16] Cosnefroy O., Murray P.J., Bishop K.N. (2016). HIV-1 capsid uncoating initiates after the first strand transfer of reverse transcription. Retrovirology.

[17] Rankovic S., Varadarajan J., Ramalho R., Aiken C., Rousso I. (2017). Reverse Transcription Mechanically Initiates HIV-1 Capsid Disassembly. J. Virol..

[18] Mamede J.I., Cianci G.C., Anderson M.R., Hope T.J. (2017). Early cytoplasmic uncoating is associated with infectivity of HIV-1. Proc. Natl. Acad. Sci. USA.

[19] Brown P.O., Bowerman B., Varmus H.E., Bishop J.M. (1987). Correct integration of retroviral DNA in vitro. Cell.

[20] Brown P.O., Bowerman B., Varmus H.E., Bishop J.M. (1989). Retroviral integration: Structure of the initial covalent product and its precursor, and a role for the viral IN protein. Proc. Natl. Acad. Sci. USA.

[21] Iordanskiy S., Berro R., Altieri M., Kashanchi F., Bukrinsky M. (2006). Intracytoplasmic maturation of the human immunodeficiency virus type 1 reverse transcription complexes determines their capacity to integrate into chromatin. Retrovirology.

[22] Balasubramaniam M., Davids B., Addai A.B., Pandhare J., Dash C. (2017). Measurement of In Vitro Integration Activity of HIV-1 Preintegration Complexes. J. Vis. Exp..

[23] McDonald D., Vodicka M.A., Lucero G., Svitkina T.M., Borisy G.G., Emerman M., Hope T.J. (2002). Visualization of the intracellular behavior of HIV in living cells. J. Cell Biol..

[24] Rasaiyaah J., Tan C.P., Fletcher A.J., Price A.J., Blondeau C., Hilditch L., Jacques D.A., Selwood D.L., James L.C., Noursadeghi M. (2013). HIV-1 evades innate immune recognition through specific cofactor recruitment. Nature.

[25] Lahaye X., Satoh T., Gentili M., Cerboni S., Conrad C., Hurbain I., El Marjou A., Lacabaratz C., Lelievre J.D., Manel N. (2013). The capsids of HIV-1 and HIV-2 determine immune detection of the viral cDNA by the innate sensor cGAS in dendritic cells. Immunity.

[26] Le Sage V., Mouland A.J., Valiente-Echeverria F. (2014). Roles of HIV-1 capsid in viral replication and immune evasion. Virus Res..

[27] Gao D., Wu J., Wu Y.T., Du F., Aroh C., Yan N., Sun L., Chen Z.J. (2013). Cyclic GMP-AMP synthase is an innate immune sensor of HIV and other retroviruses. Science.

[28] Forshey B.M., von Schwedler U., Sundquist W.I., Aiken C. (2002). Formation of a human immunodeficiency virus type 1 core of optimal stability is crucial for viral replication. J. Virol..

[29] von Schwedler U.K., Stray K.M., Garrus J.E., Sundquist W.I. (2003). Functional surfaces of the human immunodeficiency virus type 1 capsid protein. J. Virol..

[30] Burdick R.C., Li C., Munshi M., Rawson J.M.O., Nagashima K., Hu W.S., Pathak V.K. (2020). HIV-1 uncoats in the nucleus near sites of integration. Proc. Natl. Acad. Sci. USA.

[31] Dharan A., Bachmann N., Talley S., Zwikelmaier V., Campbell E.M. (2020). Nuclear pore blockade reveals that HIV-1 completes reverse transcription and uncoating in the nucleus. Nat. Microbiol..

[32] Selyutina A., Persaud M., Lee K., KewalRamani V., Diaz-Griffero F. (2020). Nuclear Import of the HIV-1 Core Precedes Reverse Transcription and Uncoating. Cell Rep..

[33] Yamashita M., Emerman M. (2004). Capsid is a dominant determinant of retrovirus infectivity in nondividing cells. J. Virol..

[34] Yamashita M., Perez O., Hope T.J., Emerman M. (2007). Evidence for direct involvement of the capsid protein in HIV infection of nondividing cells. PLoS Pathog..

[35] Lin D.H., Hoelz A. (2019). The Structure of the Nuclear Pore Complex (An Update). Annu. Rev. Biochem..

[36] Zila V., Margiotta E., Turonova B., Muller T.G., Zimmerli C.E., Mattei S., Allegretti M., Borner K., Rada J., Muller B. (2021). Cone-shaped HIV-1 capsids are transported through intact nuclear pores. Cell.

[37] von Appen A., Kosinski J., Sparks L., Ori A., DiGuilio A.L., Vollmer B., Mackmull M.T., Banterle N., Parca L., Kastritis P. (2015). In situ structural analysis of the human nuclear pore complex. Nature.

[38] Dismuke D.J., Aiken C. (2006). Evidence for a functional link between uncoating of the human immunodeficiency virus type 1 core and nuclear import of the viral preintegration complex. J. Virol..

[39] Arhel N.J., Souquere-Besse S., Munier S., Souque P., Guadagnini S., Rutherford S., Prevost M.C., Allen T.D., Charneau P. (2007). HIV-1 DNA Flap formation promotes uncoating of the pre-integration complex at the nuclear pore. EMBO J..

[40] Burdick R.C., Hu W.S., Pathak V.K. (2013). Nuclear import of APOBEC3F-labeled HIV-1 preintegration complexes. Proc. Natl. Acad. Sci. USA.

[41] Francis A.C., Marin M., Shi J., Aiken C., Melikyan G.B. (2016). Time-Resolved Imaging of Single HIV-1 Uncoating In Vitro and in Living Cells. PLoS Pathog..

[42] Francis A.C., Melikyan G.B. (2018). Single HIV-1 Imaging Reveals Progression of Infection through CA-Dependent Steps of Docking at the Nuclear Pore, Uncoating, and Nuclear Transport. Cell Host Microbe.

[43] Francis A.C., Marin M., Singh P.K., Achuthan V., Prellberg M.J., Palermino-Rowland K., Lan S., Tedbury P.R., Sarafianos S.G., Engelman A.N. (2020). HIV-1 replication complexes accumulate in nuclear speckles and integrate into speckle-associated genomic domains. Nat. Commun..

[44] Francis A.C., Marin M., Prellberg M.J., Palermino-Rowland K., Melikyan G.B. (2020). HIV-1 Uncoating and Nuclear Import Precede the Completion of Reverse Transcription in Cell Lines and in Primary Macrophages. Viruses.

[45] Price A.J., Fletcher A.J., Schaller T., Elliott T., Lee K., KewalRamani V.N., Chin J.W., Towers G.J., James L.C. (2012). CPSF6 defines a conserved capsid interface that modulates HIV-1 replication. PLoS Pathog..

[46] Price A.J., Jacques D.A., McEwan W.A., Fletcher A.J., Essig S., Chin J.W., Halambage U.D., Aiken C., James L.C. (2014). Host cofactors and pharmacologic ligands share an essential interface in HIV-1 capsid that is lost upon disassembly. PLoS Pathog..

[47] Bhattacharya A., Alam S.L., Fricke T., Zadrozny K., Sedzicki J., Taylor A.B., Demeler B., Pornillos O., Ganser-Pornillos B.K., Diaz-Griffero F. (2014). Structural basis of HIV-1 capsid recognition by PF74 and CPSF6. Proc. Natl. Acad. Sci. USA.

[48] Bejarano D.A., Peng K., Laketa V., Borner K., Jost K.L., Lucic B., Glass B., Lusic M., Muller B., Krausslich H.G. (2019). HIV-1 nuclear import in macrophages is regulated by CPSF6-capsid interactions at the nuclear pore complex. Elife.

[49] Chin C.R., Perreira J.M., Savidis G., Portmann J.M., Aker A.M., Feeley E.M., Smith M.C., Brass A.L. (2015). Direct Visualization of HIV-1 Replication Intermediates Shows that Capsid and CPSF6 Modulate HIV-1 Intra-nuclear Invasion and Integration. Cell Rep..

[50] Achuthan V., Perreira J.M., Sowd G.A., Puray-Chavez M., McDougall W.M., Paulucci-Holthauzen A., Wu X., Fadel H.J., Poeschla E.M., Multani A.S. (2018). Capsid-CPSF6 Interaction Licenses Nuclear HIV-1 Trafficking to Sites of Viral DNA Integration. Cell Host Microbe.

[51] Achuthan V., Perreira J.M., Ahn J.J., Brass A.L., Engelman A.N. (2019). Capsid-CPSF6 interaction: Master regulator of nuclear HIV-1 positioning and integration. J. Life Sci..

[52] Li W., Singh P.K., Sowd G.A., Bedwell G.J., Jang S., Achuthan V., Oleru A.V., Wong D., Fadel H.J., Lee K. (2020). CPSF6-Dependent Targeting of Speckle-Associated Domains Distinguishes Primate from Nonprimate Lentiviral Integration. mBio.

[53] Beck M., Baumeister W. (2016). Cryo-Electron Tomography: Can it Reveal the Molecular Sociology of Cells in Atomic Detail?. Trends Cell Biol..

[54] Zimmerli C.E., Allegretti M., Rantos V., Goetz S.K., Obarska-Kosinska A., Zagoriy I., Halavatyi A., Mahamid J., Kosinski J., Beck M. (2020). Nuclear pores constrict upon energy depletion. bioRxiv.

[55] Muller T.G., Zila V., Peters K., Schifferdecker S., Stanic M., Lucic B., Laketa V., Lusic M., Muller B., Krausslich H.G. (2021). HIV-1 uncoating by release of viral cDNA from capsid-like structures in the nucleus of infected cells. Elife.

[56] Blanco-Rodriguez G., Gazi A., Monel B., Frabetti S., Scoca V., Mueller F., Schwartz O., Krijnse-Locker J., Charneau P., Di Nunzio F. (2020). Remodeling of the Core Leads HIV-1 Preintegration Complex into the Nucleus of Human Lymphocytes. J. Virol..

[57] Guedán A., Donaldson C.D., Cosnefroy O., Taylor I.A., Bishop K.N. (2021). HIV-1 requires capsid remodelling at the nuclear pore for nuclear entry and integration. bioRxiv.

[58] Li C., Burdick R.C., Nagashima K., Hu W.S., Pathak V.K. (2021). HIV-1 cores retain their integrity until minutes before uncoating in the nucleus. Proc. Natl. Acad. Sci. USA.

[59] Peng K., Muranyi W., Glass B., Laketa V., Yant S.R., Tsai L., Cihlar T., Muller B., Krausslich H.G. (2014). Quantitative microscopy of functional HIV post-entry complexes reveals association of replication with the viral capsid. Elife.

[60] Bejarano D.A., Puertas M.C., Borner K., Martinez-Picado J., Muller B., Krausslich H.G. (2018). Detailed Characterization of Early HIV-1 Replication Dynamics in Primary Human Macrophages. Viruses.

[61] Zurnic Bonisch I., Dirix L., Lemmens V., Borrenberghs D., De Wit F., Vernaillen F., Rocha S., Christ F., Hendrix J., Hofkens J. (2020). Capsid-Labelled HIV To Investigate the Role of Capsid during Nuclear Import and Integration. J. Virol..

[62] Rankovic S., Deshpande A., Harel S., Aiken C., Rousso I. (2021). HIV-1 uncoating occurs via a series of rapid biomechanical changes in the core related to individual stages of reverse transcription. J. Virol..

[63] Christensen D.E., Ganser-Pornillos B.K., Johnson J.S., Pornillos O., Sundquist W.I. (2020). Reconstitution and visualization of HIV-1 capsid-dependent replication and integration in vitro. Science.

[64] Scoca V., Louveaux M., Morin R., Ershov D., Tinevez J., di Nunzio F. (2020). Direct tracking of single proviruses reveals HIV-1/LEDGF complexes excluded from virus-induced membraneless organelles. bioRxiv.

[65] Rensen E., Mueller F., Scoca V., Parmar J.J., Souque P., Zimmer C., Di Nunzio F. (2021). Clustering and reverse transcription of HIV-1 genomes in nuclear niches of macrophages. EMBO J..

[66] Sabo Y., Walsh D., Barry D.S., Tinaztepe S., de Los Santos K., Goff S.P., Gundersen G.G., Naghavi M.H. (2013). HIV-1 induces the formation of stable microtubules to enhance early infection. Cell Host Microbe.

[67] Lukic Z., Dharan A., Fricke T., Diaz-Griffero F., Campbell E.M. (2014). HIV-1 uncoating is facilitated by dynein and kinesin 1. J. Virol..

[68] Malikov V., da Silva E.S., Jovasevic V., Bennett G., de Souza Aranha Vieira D.A., Schulte B., Diaz-Griffero F., Walsh D., Naghavi M.H. (2015). HIV-1 capsids bind and exploit the kinesin-1 adaptor FEZ1 for inward movement to the nucleus. Nat. Commun..

[69] Huang P.T., Summers B.J., Xu C., Perilla J.R., Malikov V., Naghavi M.H., Xiong Y. (2019). FEZ1 Is Recruited to a Conserved Cofactor Site on Capsid to Promote HIV-1 Trafficking. Cell Rep..

[70] Gamble T.R., Vajdos F.F., Yoo S., Worthylake D.K., Houseweart M., Sundquist W.I., Hill C.P. (1996). Crystal structure of human cyclophilin A bound to the amino-terminal domain of HIV-1 capsid. Cell.

[71] Hatziioannou T., Perez-Caballero D., Cowan S., Bieniasz P.D. (2005). Cyclophilin interactions with incoming human immunodeficiency virus type 1 capsids with opposing effects on infectivity in human cells. J. Virol..

[72] Schaller T., Ocwieja K.E., Rasaiyaah J., Price A.J., Brady T.L., Roth S.L., Hue S., Fletcher A.J., Lee K., KewalRamani V.N. (2011). HIV-1 capsid-cyclophilin interactions determine nuclear import pathway, integration targeting and replication efficiency. PLoS Pathog..

[73] De Iaco A., Luban J. (2014). Cyclophilin A promotes HIV-1 reverse transcription but its effect on transduction correlates best with its effect on nuclear entry of viral cDNA. Retrovirology.

[74] Stremlau M., Owens C.M., Perron M.J., Kiessling M., Autissier P., Sodroski J. (2004). The cytoplasmic body component TRIM5alpha restricts HIV-1 infection in Old World monkeys. Nature.

[75] Liu Z., Pan Q., Ding S., Qian J., Xu F., Zhou J., Cen S., Guo F., Liang C. (2013). The interferon-inducible MxB protein inhibits HIV-1 infection. Cell Host Microbe.

[76] Goujon C., Moncorge O., Bauby H., Doyle T., Ward C.C., Schaller T., Hue S., Barclay W.S., Schulz R., Malim M.H. (2013). Human MX2 is an interferon-induced post-entry inhibitor of HIV-1 infection. Nature.

[77] Kane M., Yadav S.S., Bitzegeio J., Kutluay S.B., Zang T., Wilson S.J., Schoggins J.W., Rice C.M., Yamashita M., Hatziioannou T. (2013). MX2 is an interferon-induced inhibitor of HIV-1 infection. Nature.

[78] Schaller T., Bulli L., Pollpeter D., Betancor G., Kutzner J., Apolonia L., Herold N., Burk R., Malim M.H. (2017). Effects of Inner Nuclear Membrane Proteins SUN1/UNC-84A and SUN2/UNC-84B on the Early Steps of HIV-1 Infection. J. Virol..

[79] Arhel N., Genovesio A., Kim K.A., Miko S., Perret E., Olivo-Marin J.C., Shorte S., Charneau P. (2006). Quantitative four-dimensional tracking of cytoplasmic and nuclear HIV-1 complexes. Nat. Methods.

[80] Fernandez J., Portilho D.M., Danckaert A., Munier S., Becker A., Roux P., Zambo A., Shorte S., Jacob Y., Vidalain P.O. (2015). Microtubule-associated proteins 1 (MAP1) promote human immunodeficiency virus type I (HIV-1) intracytoplasmic routing to the nucleus. J. Biol. Chem..

[81] Dharan A., Talley S., Tripathi A., Mamede J.I., Majetschak M., Hope T.J., Campbell E.M. (2016). KIF5B and Nup358 Cooperatively Mediate the Nuclear Import of HIV-1 during Infection. PLoS Pathog..

[82] Carnes S.K., Zhou J., Aiken C. (2018). HIV-1 Engages a Dynein-Dynactin-BICD2 Complex for Infection and Transport to the Nucleus. J. Virol..

[83] Dharan A., Opp S., Abdel-Rahim O., Keceli S.K., Imam S., Diaz-Griffero F., Campbell E.M. (2017). Bicaudal D2 facilitates the cytoplasmic trafficking and nuclear import of HIV-1 genomes during infection. Proc. Natl. Acad. Sci. USA.

[84] Li Y., Kar A.K., Sodroski J. (2009). Target cell type-dependent modulation of human immunodeficiency virus type 1 capsid disassembly by cyclophilin A. J. Virol..

[85] Shah V.B., Shi J., Hout D.R., Oztop I., Krishnan L., Ahn J., Shotwell M.S., Engelman A., Aiken C. (2013). The host proteins transportin SR2/TNPO3 and cyclophilin A exert opposing effects on HIV-1 uncoating. J. Virol..

[86] Fricke T., Brandariz-Nunez A., Wang X., Smith A.B., Diaz-Griffero F. (2013). Human cytosolic extracts stabilize the HIV-1 core. J. Virol..

[87] Liu C., Perilla J.R., Ning J., Lu M., Hou G., Ramalho R., Himes B.A., Zhao G., Bedwell G.J., Byeon I.J. (2016). Cyclophilin A stabilizes the HIV-1 capsid through a novel non-canonical binding site. Nat. Commun..

[88] Ni T., Gerard S., Zhao G., Dent K., Ning J., Zhou J., Shi J., Anderson-Daniels J., Li W., Jang S. (2020). Intrinsic curvature of the HIV-1 CA hexamer underlies capsid topology and interaction with cyclophilin A. Nat. Struct. Mol. Biol..

[89] Franke E.K., Yuan H.E., Luban J. (1994). Specific incorporation of cyclophilin A into HIV-1 virions. Nature.

[90] Braaten D., Luban J. (2001). Cyclophilin A regulates HIV-1 infectivity, as demonstrated by gene targeting in human T cells. EMBO J..

[91] Wiegers K., Rutter G., Schubert U., Grattinger M., Krausslich H.G. (1999). Cyclophilin A incorporation is not required for human immunodeficiency virus type 1 particle maturation and does not destabilize the mature capsid. Virology.

[92] Sokolskaja E., Sayah D.M., Luban J. (2004). Target cell cyclophilin A modulates human immunodeficiency virus type 1 infectivity. J. Virol..

[93] Kim K., Dauphin A., Komurlu S., McCauley S.M., Yurkovetskiy L., Carbone C., Diehl W.E., Strambio-De-Castillia C., Campbell E.M., Luban J. (2019). Cyclophilin A protects HIV-1 from restriction by human TRIM5alpha. Nat. Microbiol..

[94] Selyutina A., Persaud M., Simons L.M., Bulnes-Ramos A., Buffone C., Martinez-Lopez A., Scoca V., Di Nunzio F., Hiatt J., Marson A. (2020). Cyclophilin A Prevents HIV-1 Restriction in Lymphocytes by Blocking Human TRIM5alpha Binding to the Viral Core. Cell Rep..

[95] Lee K., Ambrose Z., Martin T.D., Oztop I., Mulky A., Julias J.G., Vandegraaff N., Baumann J.G., Wang R., Yuen W. (2010). Flexible use of nuclear import pathways by HIV-1. Cell Host Microbe.

[96] Matreyek K.A., Engelman A. (2011). The requirement for nucleoporin NUP153 during human immunodeficiency virus type 1 infection is determined by the viral capsid. J. Virol..

[97] Kane M., Rebensburg S.V., Takata M.A., Zang T.M., Yamashita M., Kvaratskhelia M., Bieniasz P.D. (2018). Nuclear pore heterogeneity influences HIV-1 infection and the antiviral activity of MX2. Elife.

[98] Burse M., Shi J., Aiken C. (2017). Cyclophilin A potentiates TRIM5alpha inhibition of HIV-1 nuclear import without promoting TRIM5alpha binding to the viral capsid. PLoS ONE.

[99] Brass A.L., Dykxhoorn D.M., Benita Y., Yan N., Engelman A., Xavier R.J., Lieberman J., Elledge S.J. (2008). Identification of host proteins required for HIV infection through a functional genomic screen. Science.

[100] Konig R., Zhou Y., Elleder D., Diamond T.L., Bonamy G.M., Irelan J.T., Chiang C.Y., Tu B.P., De Jesus P.D., Lilley C.E. (2008). Global analysis of host-pathogen interactions that regulate early-stage HIV-1 replication. Cell.

[101] Krishnan L., Matreyek K.A., Oztop I., Lee K., Tipper C.H., Li X., Dar M.J., Kewalramani V.N., Engelman A. (2010). The requirement for cellular transportin 3 (TNPO3 or TRN-SR2) during infection maps to human immunodeficiency virus type 1 capsid and not integrase. J. Virol..

[102] Zhou L., Sokolskaja E., Jolly C., James W., Cowley S.A., Fassati A. (2011). Transportin 3 promotes a nuclear maturation step required for efficient HIV-1 integration. PLoS Pathog..

[103] De Iaco A., Santoni F., Vannier A., Guipponi M., Antonarakis S., Luban J. (2013). TNPO3 protects HIV-1 replication from CPSF6-mediated capsid stabilization in the host cell cytoplasm. Retrovirology.

[104] Christ F., Thys W., De Rijck J., Gijsbers R., Albanese A., Arosio D., Emiliani S., Rain J.C., Benarous R., Cereseto A. (2008). Transportin-SR2 imports HIV into the nucleus. Curr. Biol..

[105] Maertens G.N., Cook N.J., Wang W., Hare S., Gupta S.S., Oztop I., Lee K., Pye V.E., Cosnefroy O., Snijders A.P. (2014). Structural basis for nuclear import of splicing factors by human Transportin 3. Proc. Natl. Acad. Sci. USA.

[106] Tabasi M., Nombela I., Janssens J., Lahousse A.P., Christ F., Debyser Z. (2021). Role of Transportin-SR2 in HIV-1 Nuclear Import. Viruses.

[107] Fernandez J., Machado A.K., Lyonnais S., Chamontin C., Gartner K., Leger T., Henriquet C., Garcia C., Portilho D.M., Pugniere M. (2019). Transportin-1 binds to the HIV-1 capsid via a nuclear localization signal and triggers uncoating. Nat. Microbiol..

[108] Zhou H., Xu M., Huang Q., Gates A.T., Zhang X.D., Castle J.C., Stec E., Ferrer M., Strulovici B., Hazuda D.J. (2008). Genome-scale RNAi screen for host factors required for HIV replication. Cell Host Microbe.

[109] Yeung M.L., Houzet L., Yedavalli V.S., Jeang K.T. (2009). A genome-wide short hairpin RNA screening of jurkat T-cells for human proteins contributing to productive HIV-1 replication. J. Biol. Chem..

[110] Di Nunzio F., Danckaert A., Fricke T., Perez P., Fernandez J., Perret E., Roux P., Shorte S., Charneau P., Diaz-Griffero F. (2012). Human nucleoporins promote HIV-1 docking at the nuclear pore, nuclear import and integration. PLoS ONE.

[111] Di Nunzio F., Fricke T., Miccio A., Valle-Casuso J.C., Perez P., Souque P., Rizzi E., Severgnini M., Mavilio F., Charneau P. (2013). Nup153 and Nup98 bind the HIV-1 core and contribute to the early steps of HIV-1 replication. Virology.

[112] Wu J., Matunis M.J., Kraemer D., Blobel G., Coutavas E. (1995). Nup358, a cytoplasmically exposed nucleoporin with peptide repeats, Ran-GTP binding sites, zinc fingers, a cyclophilin A homologous domain, and a leucine-rich region. J. Biol. Chem..

[113] Wilken N., Senecal J.L., Scheer U., Dabauvalle M.C. (1995). Localization of the Ran-GTP binding protein RanBP2 at the cytoplasmic side of the nuclear pore complex. Eur. J. Cell Biol..

[114] Bichel K., Price A.J., Schaller T., Towers G.J., Freund S.M., James L.C. (2013). HIV-1 capsid undergoes coupled binding and isomerization by the nuclear pore protein NUP358. Retrovirology.

[115] Sukegawa J., Blobel G. (1993). A nuclear pore complex protein that contains zinc finger motifs, binds DNA, and faces the nucleoplasm. Cell.

[116] Bastos R., Lin A., Enarson M., Burke B. (1996). Targeting and function in mRNA export of nuclear pore complex protein Nup153. J. Cell Biol..

[117] Matreyek K.A., Yucel S.S., Li X., Engelman A. (2013). Nucleoporin NUP153 phenylalanine-glycine motifs engage a common binding pocket within the HIV-1 capsid protein to mediate lentiviral infectivity. PLoS Pathog..

[118] Zhong Z., Ning J., Boggs E.A., Jang S., Wallace C., Telmer C., Bruchez M.P., Ahn J., Engelman A.N., Zhang P. (2021). Cytoplasmic CPSF6 Regulates HIV-1 Capsid Trafficking and Infection in a Cyclophilin A-Dependent Manner. mBio.

[119] Rankovic S., Ramalho R., Aiken C., Rousso I. (2018). PF74 Reinforces the HIV-1 Capsid To Impair Reverse Transcription-Induced Uncoating. J. Virol..

[120] Yant S.R., Mulato A., Hansen D., Tse W.C., Niedziela-Majka A., Zhang J.R., Stepan G.J., Jin D., Wong M.H., Perreira J.M. (2019). A highly potent long-acting small-molecule HIV-1 capsid inhibitor with efficacy in a humanized mouse model. Nat. Med..

[121] Ester S.M., Wei G., Zhao H., Adu-Ampratwum D., Iqbal N., Courouble V.V., Francis A.C., Annamalai A.S., Singh P.K., Shkriabai N. (2020). Structural and mechanistic bases for a potent HIV-1 capsid inhibitor. Science.

[122] Zhuang S., Torbett B.E. (2021). Interactions of HIV-1 Capsid with Host Factors and Their Implications for Developing Novel Therapeutics. Viruses.

